# GABA treatment does not induce neogenesis of new endocrine cells from pancreatic ductal cells

**DOI:** 10.1080/19382014.2023.2219477

**Published:** 2023-05-31

**Authors:** Shihao Wang, Xin Dong, Mahan Maazi, Nan Chen, Amar Mahil, Janel L. Kopp

**Affiliations:** Department of Cellular and Physiological Sciences, Life Sciences Institute, University of British Columbia, Vancouver, BC, Canada

**Keywords:** alpha cells, alpha-to-beta cell transdifferentiation, beta cells, ductal cells, endocrine neogenesis, GABA

## Abstract

Previous studies indicated that ductal cells can contribute to endocrine neogenesis in adult rodents after alpha cells convert into beta cells. This can occur through Pax4 mis-expression in alpha cells or through long-term administration of gamma-aminobutyric acid (GABA) to healthy mice. GABA has also been reported to increase the number of beta cells through direct effects on their proliferation, but only in specific genetic mouse backgrounds. To test whether GABA induces neogenesis of beta cells from ductal cells or affects pancreatic cell proliferation, we administered GABA or saline over 2 or 6 months to *Sox9CreER;R26R^YFP^* mice in which 60–80% of large or small ducts were efficiently lineage labeled. We did not observe any increases in islet neogenesis from ductal cells between 1 and 2 months of age in saline treated mice, nor between 2 and 6 months of saline treatment, supporting previous studies indicating that adult ductal cells do not give rise to new endocrine cells during homeostasis. Unlike previous reports, we did not observe an increase in beta cell neogenesis after 2 or 6 months of GABA administration. Nor did we observe a significant increase in the pancreatic islet area, the number of insulin and glucagon double positive cells, or cell proliferation in the pancreas. This indicates that the effect of long term GABA administration on the pancreas is minimal or highly context dependent.

## GRAPHICAL ABSTRACT

**Figure:**
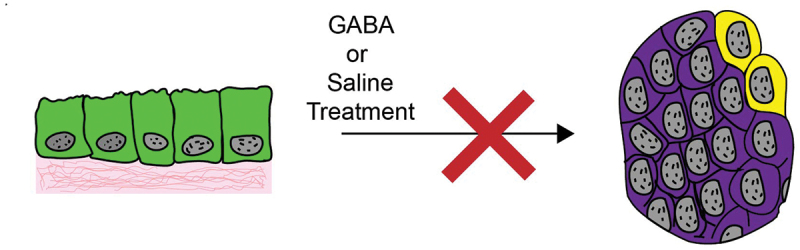

## Introduction

Pancreatic beta cells are lost through autoimmune-mediated destruction during the development of Type I diabetes. Efforts to replace these lost cells by potentially generating new beta cells from a source of endogenous cells have focused on increasing replication of existing beta cells or identifying potential non-beta cell sources, such as alpha, delta, acinar, or ductal cells.^1–12^ A promising method of generating new beta cells by inducing alpha cells to transdifferentiate into beta cells via transgenic Pax4 expression was suggested to be accompanied by generation of new alpha cells from ductal cells.^1,3^ Subsequently, it was discovered that gamma-aminobutyric acid (GABA) signaling could also increase beta cells and decrease alpha cells in mouse models and human islets^1^ and this raised the exciting possibility that it might be possible to increase beta cell numbers in patients using this method. In fact, a clinical trial was registered in 2018 to test the effects of long-term GABA administration in persons living with Type I diabetes, an indication of the excitement in the field at the potential of this molecule.

GABA is a neurotransmitter released at synaptic terminals to inhibit neuronal firing in the central nervous system.^13^ GABA is also present in the pancreatic islets, but the source of GABA and its role in islet function is somewhat unclear.^14,15^ Previous studies have reported that GABA can affect glucagon secretion,^16,17^ Arx expression,^1,16^ as well as promote increased beta cell mass.^1,16^ The GABA-induced expansion of beta cell mass has been attributed to beta cell proliferation^16^ and/or alpha cell transdifferentiation into beta cells.^1^ However, these results appear to be highly context dependent.^16,18,19^ Specifically, beta cell mass increases in response to GABA treatment have been reported to only occur in CD1 background mice, and not in Bl6 background mice, and only occur in CD1 mice fed normal chow diet compared to high fat diet.^16^ Additionally, other studies also found that Bl6 mice exposed to GABA failed to increase beta cell area and no alpha-to-beta cell transdifferentiation was observed using a *Glucagon-CreER* lineage tracing mouse model.^18^ Finally, collaborative efforts to recapitulate these studies with the original study parameters replicated as closely as possible demonstrated that the effects of GABA may not be widely reproducible.^19^ Altogether, these subsequent validation studies suggested that GABA-induced generation of beta cells from alpha cells may be dependent on the background of mice or particular housing conditions. Additionally, whether alpha cells can be generated from large pancreatic ducts in the presence of GABA^1^ has not been tested by other groups.

In this study, we used two different transgenic founder lines of the *Sox9CreER* mouse line to specifically lineage label ductal cells using the *R26R*^*YFP*^ allele with high efficiency in a predominantly CD1 background. We then treated these *Sox9CreER,R26R*^*YFP*^ mice with saline or GABA for 2 months or 6 months and examined whether beta cell mass expanded or ductal cells could form beta cells as predicted by the original studies.^1^ In our studies, we found no evidence that long-term GABA administration in adult mice induced ductal cells to transdifferentiate into beta cells.

## Results

### *Sox9^+^ cells in large ducts were efficiently labeled in 34.1* Sox9CreER;R26R^YFP^ *mice*

**Figure 1.**
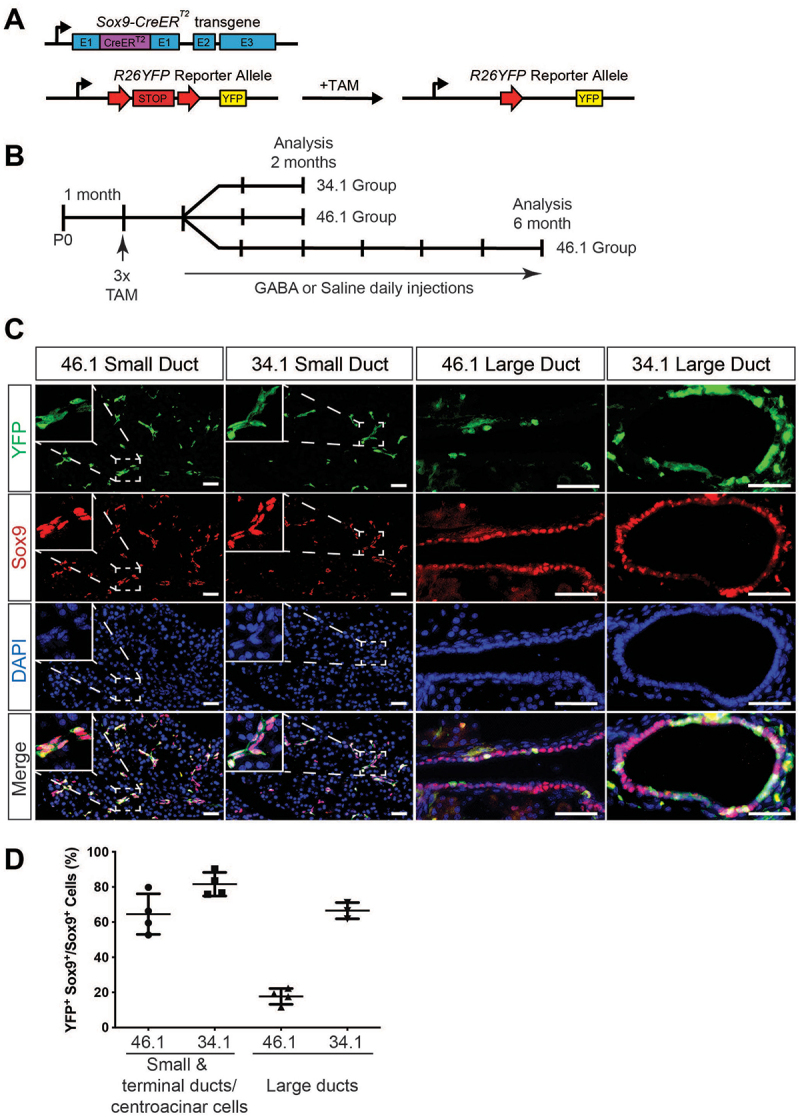
Unique *Sox9CreER* transgenic founders label large pancreatic ducts with different efficiencies. (A) *Sox9CreER;R26R^YFP^* mice were injected with tamoxifen (TAM) to induce Cre-mediated recombination of the floxed STOP cassette in the *Rosa26* locus, resulting in YFP expression in ductal cells. (B) One-month-old *Sox9CreER;R26R^YFP^* mice were injected daily with TAM for three consecutive days. After one month, mice were randomly selected for daily IP injection of GABA or saline for two (2 M–46.1 and 34.1 cohorts) or six (6 M–46.1 cohort only) months. All mice were injected intraperitoneally with EdU daily for 10 days prior to dissection. (C) Representative immunofluorescent images from big or small ducts of YFP labeled Sox9^+^ ductal cells for *Sox9CreER;R26R^YFP^* mice of the 34.1 or 46.1 founder lineage. (D) Quantification of the number of YFP^+^Sox9^+^ cells relative to the total Sox9^+^ cells in small or large diameter ducts. Scale bar: 40 µm (C).

To trace adult Sox9^+^ ductal cells, *Sox9CreER;R26R*^*YFP*^ mice from the 46.1 founder lineage,^20,21^ which has lower recombination frequency but no recombination in the absence of tamoxifen,^20^ were injected with tamoxifen (TAM) at 1 month of age (*n* = 22, Figure 1A,B). We harvested pancreata from four mice after one month of TAM administration and stained them with anti-Sox9 and anti-green fluorescent protein (GFP) antibodies to evaluate the ductal cell labeling efficiency with YFP (Figure 1C). We found that an average of 64.58 ± 11.57% of the Sox9^+^ cells in small ducts were labeled with YFP, but significantly fewer Sox9^+^ cells were labeled with YFP in the large ducts of this founder (Figure 1D). To examine whether the large ducts also contribute to alpha and beta cell neogenesis with GABA treatment, we utilized the 34.1 *Sox9CreER* founder^22–24^ to create *Sox9CreER;R26R*^*YFP*^ mice. This founder has some recombination in the absence of tamoxifen,^24^ but has a much higher recombination rate of Sox9^+^ cells. When we injected these 34.1 *Sox9CreER;R26R*^*YFP*^ mice with TAM at 1 month (*n* = 12, Figure 1A,B) and quantified the labeling efficiency in big and small ducts in four of them, ~80% of the Sox9^+^ cells in small ducts and 66.50 ± 4.60% of Sox9^+^ cells in large ducts were labeled with YFP (*n* = 4, Figure 1C,D). Together, these models allow us to specifically trace the fate of both large and small ducts, including terminal ducts/centroacinar cells.

## Long-term GABA treatment did not induce beta cell neogenesis from Sox9^+^ cells

**Figure 2.**
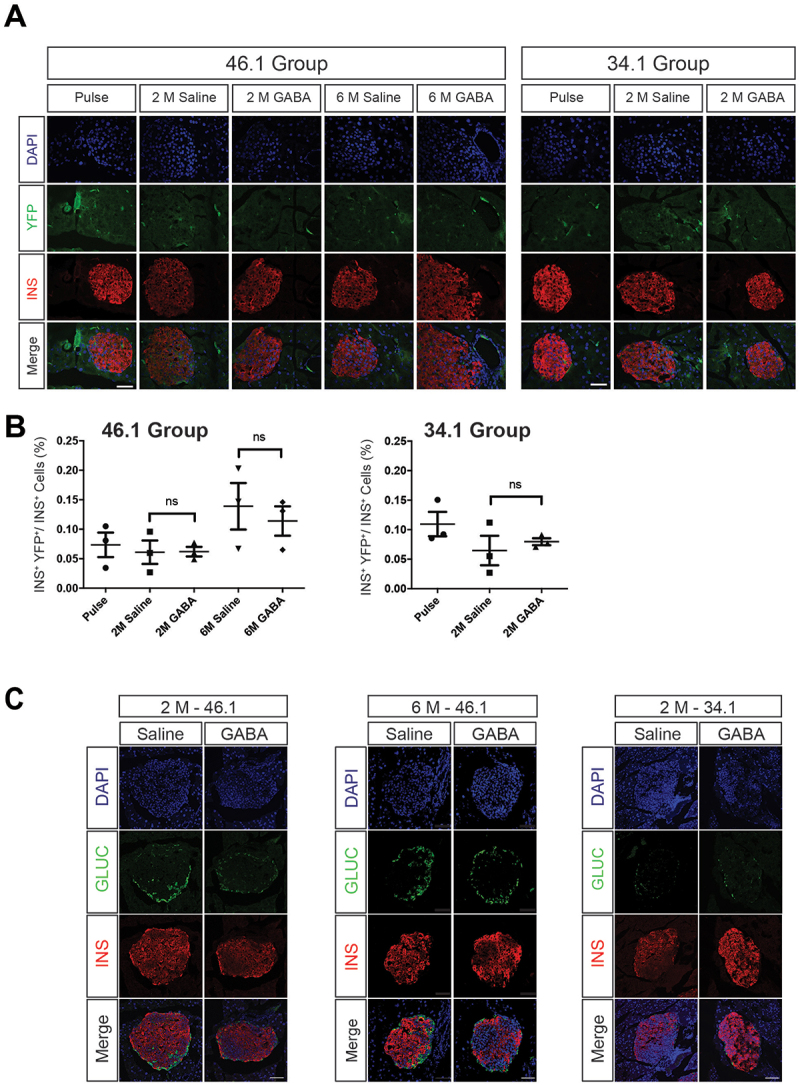
New insulin^+^ cells do not arise from Sox9^+^ cells with GABA Treatment. (A) Representative images of immunofluorescence staining for insulin and YFP. (B) Quantification of the number of insulin^+^YFP^+^ cells relative to the total number of insulin^+^ cells. Data points from individual mice are presented with mean ± SD. Bootstrap analyses were used to test for significance. No significant differences were detected. (C) Representative images of immunofluorescence staining for insulin and glucagon. No insulin and glucagon co-positive cells were found in all sections analyzed. Scale bar: 40 µm (A,C).

The remaining 46.1 and 34.1 *Sox9CreER;R26R*^*YFP*^ mice were randomly selected to receive daily GABA or saline injections for 2 months (*n* = 3–4 each treatment 46.1 and 34.1 groups) or 6 months (*n* = 3–4 each treatment for only the 46.1 group). We collected the pancreata and performed co-immunofluorescence staining for insulin and YFP (Figure 2A) on these mice, as well as those collected 1 month after tamoxifen treatment (Figure 1- labeled “Pulse” in Figure 2) to quantify the number of beta cells arising from YFP-lineage labeled ductal cells over time. Consistent with previous studies,^20^ there were a small number of YFP^+^ beta cells at the Pulse time point, but there were no significant differences in lineage labeled beta cells between the 2-month-old Pulse and the 4-month-old saline-treated 46.1 *Sox9CreER;R26R*^*YFP*^ mice (Figure 2A-B, 46.1 groups). Additionally, increasing the labeling efficiency did not alter this outcome (Figure 2A-B, 34.1 groups). There was also no significant difference in beta cell labeling after 2 months of daily GABA injections (Figure 2A-B, 2 month (M) 46.1 and 34.1 GABA groups compared to the respective saline groups). Because previous studies had also demonstrated that 6 months of GABA treatment could induce beta cell neogenesis,^1^ we also treated some 46.1 *Sox9CreER;R26R*^*YFP*^ mice for 6 months (6 M- Figure 1A-B). However, the percentage of lineage-labeled beta cells remained low in the Saline and GABA treated groups with only ~ 0.1–0.2% of beta cells being labeled by YFP (Figure 2A-B). In sum, we did not observe a substantial contribution of Sox9^+^ ductal cells to beta cells over time or with GABA treatment even when the labeling efficiency of Sox9^+^ ductal cells was quite high.

## Islet area and overall cell proliferation did not increase with GABA injection

Previous studies have questioned whether GABA treatment could induce alpha-to-beta cell transdifferentiation accompanied by compensatory ductal-to-alpha cell neogenesis.^18,19,25^ To examine whether GABA was inducing alpha-to-beta cell transdifferentiation, we performed immunofluorescence staining for glucagon and insulin, but did not observe any co-positive cells in any treatment or age (Figure 2C), suggesting that GABA treatment did not induce the alpha-to-beta cell transdifferentiation observed in previous studies.^1^

**Figure 3.**
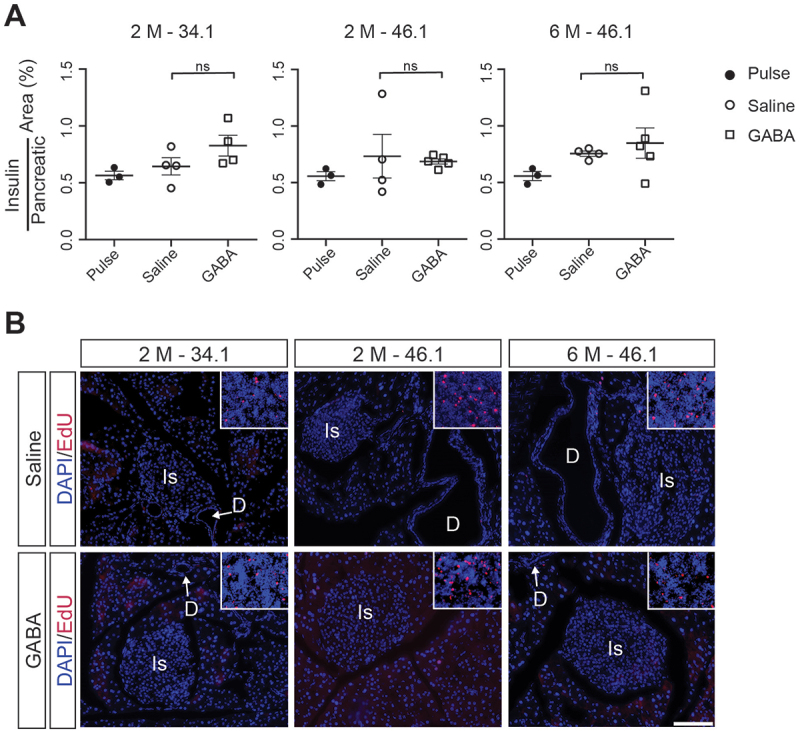
Neither insulin^+^ area nor proliferation of pancreatic cells changes in response to GABA treatment. (A) Quantification of the average insulin^+^ area over total pancreatic area. Data points from individual mice are shown and the mean ± SD are indicated. Bootstrap analyses were used to test for significance. No significant differences were detected. (B) Representative images of immunofluorescence staining for EdU^+^ cells in the pancreas. Insets: Representative images from the lymph nodes present on the same sections are included as a positive control for EdU staining. Is: Islet, D: Duct. Scale bar: 50 µm (B).

To further address whether there were any changes in beta cell area with GABA treatment, we quantified the insulin^+^ cell area using insulin immunofluorescence staining (Figures 2A and 3A). We found that there were no significant differences in insulin^+^ area over total pancreatic area in mice treated with GABA compared to the respective saline-treated control groups in either founder lineages (Figure 3A). Additionally, the overall proliferation in the pancreas, as observed by immunofluorescence staining for EdU^+^ cells in the pancreas (Figure 3B), was not different. Altogether, these data are consistent with previously published studies where no increases in endocrine cell area were observed in response to long-term GABA treatment.^18^

## Discussion

In this article, we tested whether administration of GABA could induce the differentiation of mature Sox9^+^ pancreatic ductal cells into alpha cells that became insulin expressing beta or beta-like cells. We found no increase in lineage traced (YFP^+^) ductal cells contributing to the insulin expressing cell population after short or long-term daily GABA administration to *Sox9CreER;R26R*^*YFP*^ mice. This suggests that GABA does not promote trans-differentiation of pancreatic ductal cells into endocrine cells. Moreover, our data suggest islet mass was not significantly affected by GABA treatment. These data strongly indicate that GABA has little to no effect on islets, even when the animals are predominantly of the CD1 background with a little Bl6 background. Whether the effects of GABA on beta cell proliferation^16^ could be recapitulated in a pure CD1 background or in other genetic contexts is still unclear.

A few studies have suggested that ductal cells can give rise to endocrine cells during homeostasis.^7,26^ Here we show that, similar to our previous studies,^20^ we do not see neogenesis of beta cells from Sox9^+^ ductal cells. Importantly, this was true in the context of two distinct *Sox9CreER* founder lines that have different recombination efficiencies in the large ducts, but similarly high recombination in small ducts. Both of our characterized and published founder lines efficiently label centroacinar and terminal ducts. The more efficient 34.1 *Sox9CreER* mouse model also targets large ducts more effectively and this could underlie the ability of this model to induce main duct intraductal papillary mucinous neoplasia in Kras^G12D^-expressing ductal cells with reduced *Pten* expression.^22^ Overall, our data support the conclusion that wild-type adult ductal cells do not give rise to other cell types during homeostasis.

Although we did not directly test whether ductal cells could form new alpha cells in this study, we rarely observed YFP^+^ cells in the islet of *Sox9CreER;R26R*^*YFP*^ mice and this did not appear to change with any duration of GABA treatment. In addition, we did not observe any insulin or glucagon co-positive cells, nor an increase in insulin positive area or pancreatic cell proliferation. Together, these data suggest that the previously reported increases in beta cell area due to beta cell proliferation^16^ or transdifferentiation from alpha cells to beta cells in the presence of GABA^1^ did not occur in our study. Our data are consistent with other studies performed in a different mouse genetic background.^18^ Given the excitement around the potential of GABA for persons living with diabetes, the pharmaceutical company novo nordisk^Ⓡ^ worked closely with the original authors to try to validate the effects of GABA, but despite trying to harmonize the experimental conditions as much as possible they also did not observe the GABA-mediated increases in beta cell mass from original study.^1,19^ Therefore, our study on GABA-induced neogenesis from ductal cells supports the conclusions from other groups’ efforts to recapitulate the effects of suppressing alpha cell fate by artemisinins or GABA on the transdifferentiation between endocrine cell types.^18,19,25^ Altogether, our work and others suggest that suppressing the alpha cell fate is unlikely to result in endocrine neogenesis from ductal cells outside the unique contexts of the original studies.

## Materials and methods

### Mice

All animal experiments were conducted at the University of British Columbia with the approval of the University of British Columbia Animal Care Committee in accordance with Canadian Council for Animal Care guidelines. The *Sox9CreER*^*T2*^ transgene was generated by modifying the RP23-229L12 bacterial artificial chromosome (BAC) clone by inserting the KOZAK-CreER^T2^-polyA sequence in place of the ATG start codon of the Sox9 open reading frame within exon 1.^20^ BAC DNA was injected into the pro-nucleus of fertilized CB6F2 oocytes (UC Irvine Transgenic Mouse Facility, CA, USA), as previously described.^20^ After screening, two successful founder transgenic lines, 46.1^20,21,27^ (JAX no. 018829) and 34,1^22–24^, were propagated on the CD1 background and used routinely. The *Sox9CreER* founder 46.1 has a lower recombination efficiency and little to no recombination in the absence of tamoxifen. The *Sox9CreER* founder 34.1 has a higher recombination efficiency and some rare recombination in the absence of tamoxifen. The *R26R*^*YFP*^ mice (JAX 006148, Bl6 background) were described previously.^28^ To generate *Sox9CreER;R26R*^*YFP*^ mice with the 46.1 or the 34.1 alleles, respectively, we crossed *Sox9CreER;R26R*^*YFP/YFP*^ males (with the 46.1 or 34.1 allele) with CD1 females (Charles River), all offspring with *Sox9CreER;R26R*^*YFP*/+^ genotype were kept regardless of gender, and these offspring had an approximate genetic background of >75% CD1 and <25% C57BL/6 background. Tamoxifen (Sigma, St Louis, MO) was dissolved at 20 mg/ml in corn oil (Sigma) and administered by 3 consecutive injections subcutaneously to these experimental mice (4 mg per 40 g body weight) during their 4th week of life. Mice were housed using the standard Optimice caging system at 22°C with 5 mice or less per cage. Daily intraperitoneal GABA (Sigma A5835-100 G) (250 µg per kilogram of bodyweight) or saline (similar volumes) injections were given to experimental and control mice, respectively, for 2 or 6 months. To assess cell proliferation, all mice received daily intraperitoneal injections of EdU for 10 days prior to euthanasia.

## Immunofluorescence staining

Tissues were fixed in 4% PFA for 30 minutes at 4°C, embedded in OCT and stored at −80°C, then 10 µm serial sections were collected. Dried sections were immersed in PBS pH = 7.4 for 10 minutes to remove the OCT, followed by permeabilization with 0.1% Triton X-100 PBS pH = 7.4 for 10 minutes and blocked in 0.1% Triton X-100 PBS pH = 7.4 containing 1% inactivated fetal bovine serum for 60 minutes. Primary antibodies were diluted in the blocking buffer, applied to sections using Shandon cassettes, and incubated overnight at 4°C. The primary antibodies used and their corresponding dilution factors were as follows: guinea pig polyclonal anti-insulin (1:500, Abcam-ab7842), goat anti-glucagon (1:500, SantaCruz-sc7780), goat anti-GFP (1:1000, Abcam-ab6673) or rat anti-GFP (1:1000, Dr. Hung-Ping Shih, City of Hope), rabbit anti-Sox9 (1:1000, Millipore-AB5535). EdU staining was performed using the Click-iT™ EdU Alexa Fluor™ 647 Imaging Kit (ThermoFisher). The secondary antibodies were used at a 1:1000 concentration and incubated at room temperature for 1 hour. Slides were scanned using a Pannoramic Midi II slide scanner (3DHISTECH). Islet pictures were taken using an Olympus FV-1000 confocal microscope.

## Morphometric analysis

For morphometric analyses, the entire embryonic or adult pancreas was sectioned and evenly distributed 10 µm sections throughout the organ were selected. Three sections (~1% of the pancreas) in these experimental mice were analyzed. The recombination efficiency of ductal cells by each of the *Sox9CreER* founders was quantified by dividing the number of Sox9 and YFP double positive cells by the total number of Sox9^+^ cells in three random fields of view from three sections per mouse. The possible contribution of ductal cells to the beta cell population was examined by dividing the number of insulin and YFP co-positive cells by the total number of insulin^+^ cells in all islets from three sections per mouse. Islet area was determined by dividing the insulin^+^ pixel area by the total DAPI^+^ pancreatic pixel area per section.
